# Supplementary material for: A determinate method of metrology attribute benchmark of commercial banks’ management efficiency
Source: PLoS One. 2022 Aug 4;17(8):e0272286. doi: 10.1371/journal.pone.0272286 (PMC9352002; doi:10.1371/journal.pone.0272286)
Supplement: S1 File — (DOCX) [file pone.0272286.s001.docx]

Description of experimental data sources：

| Bank | Years | Data Source Name | URL |
| --- | --- | --- | --- |
| ICBC | 2010 | 2010 Annual Report of Industrial and Commercial Bank of China Limited | http://quotes.money.163.com/f10/ggmx_601398_689197.html |
|  | 2011、2012、2013 | 2013 ICBC Annual Report | https://wenku.baidu.com/view/ca8590c25a1b6bd97f192279168884868662b86a.html |
|  | 2014、2015、2016 | ICBC 2016 Annual Report | <https://www.docin.com/p-1881527613.html> |
| Bank of Communications | 2010 | 2010 Bank of Communications Annual Report | <https://www.doc88.com/p-6721893143066.html> |
|  | 2011、2012、2013 | Bank of Communications 2013 Annual Report | https://www.doc88.com/p-9999420631620.html |
|  | 2014、2015、2016 | Bank of Communications 2016 Annual Report | https://www.doc88.com/p-5774921683430.html?r=1 |
| Construction bank | 2009、2010 | China Construction Bank 2010 Annual Report | <https://www.doc88.com/p-78674706212.html> |
|  | 2011、2012、2013 | 2013 CCB Annual Report | <https://www.doc88.com/p-78674706212.html> |
|  | 2014、2015、2016 | China Construction Bank 2016 Annual Report | <https://www.docin.com/p-1880960358.html> |
| Agricultural | 2009、2010 | Agricultural Bank of China Annual Report 2010 | <https://www.doc88.com/p-7465431854083.html> |
|  | 2011、2012、2013 | Agricultural Bank of China 2013 Annual Report | <https://www.docin.com/p-1013084057.html> |
|  | 2014、2015、2016 | A Brief Analysis of the 2016 Annual Report of Agricultural Bank of China | <https://zhuanlan.zhihu.com/p/26247054> |
