# Supplementary material for: A determinate method of metrology attribute benchmark of commercial banks’ management efficiency
Source: PLoS One. 2022 Aug 4;17(8):e0272286. doi: 10.1371/journal.pone.0272286 (PMC9352002; doi:10.1371/journal.pone.0272286)
Supplement: Attachment — Submitted filename: Review Repor7.pdf [file pone.0272286.s002.pdf]

Review Report

Name of the Journal: PLOS ONE

Manuscript id: PONE-D-21-34169

Title of the manuscript: A Determinate method of“ metrology attribute benchmark of Commercial Banks" Management Efficiency

The study utilizes cluster mining technology to analyze the attribute data of several commercial banks based on mastering the management efficiency metrology attribute data, mining their characteristics and patterns, and determines the candidate benchmark set. In the candidate benchmark, the metrology attribute benchmark is determined by using average value, thus obtaining a universal metrology attribute benchmark. The study employs the statistical clustering analysis technique in the field of efficiency management .

Comments and Suggestions.

- Replace “ belongs” by “belong “ in the statement: “Let the condensed point set belongs to E” .y
- References are not uniformly written following the journal standard format. Check all the references.
