# Supplementary material for: A determinate method of metrology attribute benchmark of commercial banks’ management efficiency
Source: PLoS One. 2022 Aug 4;17(8):e0272286. doi: 10.1371/journal.pone.0272286 (PMC9352002; doi:10.1371/journal.pone.0272286)
Supplement: Attachment — Submitted filename: response-to-reviewers.docx [file pone.0272286.s003.docx]

**Original Manuscript ID:** [**PONE-D-21-34169]-[EMID:09d44c3897e2ec49]**

**Original Article Title:** A Determinate Method of Metrology Attribute Benchmark of Commercial Banks’ Management Efficiency

**To:** PLOS ONE Editor

**Re:** Response to reviewers

Dear Editor,

Thank you for giving us an opportunity to address the reviewers’ comments. We are uploading (a) our point-by-point response to the comments (below) (response to reviewers), (b) an updated manuscript with yellow highlighting indicating changes, and (c) a clean updated manuscript without highlights (PDF main document).

Best regards,

Ren Jing, Chang Xin

**Response to reviewers**

**Original Manuscript ID:** [**PONE-D-21-34169]-[EMID:09d44c3897e2ec49]**

**Original Article Title:** A Determinate Method of Metrology Attribute Benchmark of Commercial Banks’ Management Efficiency

**To:** PLOS ONE Editor

**Re:** Response to reviewers

**Reviewer#1, Concern # 1:** Replace “belongs” by “belong” in the statement: “Let the condensed point set belongs to E”.

**Author response:** We updated the manuscript by replacing “belongs” by “belong” in the statement: “Let the condensed point set belongs to E”.

**Author action:** We updated the manuscript by replacing “belongs” by “belong” in the statement: “Let the condensed point set belongs to E”. **(Page7)**

**Reviewer#1, Concern # 2:** References are not uniformly written following the journal standard format. Check all the references.

**Author response:** We have changed the reference format according to the journal requirements standard.

**Author action:** We updated the manuscript by changing the reference format according to the journal requirements standard. **(Page16-17)**

**Reviewer#2, Concern # 1:** The authors started the abstract with the aim of the study as follows: (In order to address the inadequacies of current management efficiency evaluation in commercial banks, the idea of management efficiency metrology is proposed.). In my opinion, they should start with the context and background of the topic.

**Author response:** We updated the manuscript by rewriting the abstract and adding a summary of background significance.

**Author action:** We updated the manuscript by rewriting the abstract and adding a summary of background significance. **(Page1)**

**Reviewer#2, Concern # 2:** again, reorganize the abstract to conclude:
(a) The overall purpose of the study and the research problems you investigated.
(b) The basic design of the study.
(c) Major findings or trends found as a result of the study.
(d) A brief summary of your interpretations and conclusions.

**Author response:** We updated the manuscript by rewriting

(a) The overall purpose of the study and the research problems you investigated.

(b) The basic design of the study.

(c) Major findings or trends found as a result of the study.

(d) A brief summary of interpretations and conclusions.

**Author action:** We updated the manuscript by revising the abstract and reorganizing the abstract from four aspects proposed by expert. **(Page1)**

**Reviewer#2, Concern # 3:** The overall presentation of the manuscript is confusing to the readers. The authors should summarize the study and presented it again with a simplified sequence of ideas without complexity.

**Author response:** We have read the full text and revised the introduction and further emphasized the research ideas in the introduction.

**Author action:** We updated the manuscript by revising introduction **(page3, Sec.1)**, describing and addressing our highlights and novel points in introduction.

**Reviewer#2, Concern # 4:** The results and discussion for the presented study need to be improved.

**Author response:** Aim to this concern, we have revised the Conclusions to make the conclusion more concise and to highlight the main work of this paper.

**Author action:** We updated the manuscript by rewriting the conclusion. **(Page15, Sec.4)**
